# Alcohol and ideal cardiovascular health: The Multi‐Ethnic Study of Atherosclerosis

**DOI:** 10.1002/clc.23125

**Published:** 2018-12-17

**Authors:** Oluseye Ogunmoroti, Olatokunbo Osibogun, Robyn L. McClelland, Gregory L. Burke, Khurram Nasir, Erin D. Michos

**Affiliations:** ^1^ The Johns Hopkins Ciccarone Center for the Prevention of Cardiovascular Disease Baltimore Maryland; ^2^ Department of Epidemiology, Robert Stempel College of Public Health and Social Work Florida International University Miami Florida; ^3^ Department of Biostatistics University of Washington Seattle Washington; ^4^ Division of Public Health Sciences Wake Forest School of Medicine Winston‐Salem North Carolina; ^5^ Center for Outcomes Research and Evaluation Yale New Haven Hospital New Haven Connecticut; ^6^ Section of Cardiovascular Medicine Yale University New Haven Connecticut

**Keywords:** alcohol consumption, ideal cardiovascular health metrics, Life's Simple 7

## Abstract

**Background:**

Alcohol consumption is associated with cardiovascular disease (CVD), with moderate drinkers having decreased CVD risk compared to non‐ and heavy drinkers. However, whether alcohol consumption is associated with ideal cardiovascular health (CVH), assessed by the American Heart Association's (AHA) Life's Simple 7 (LS7) metrics, and whether associations differ by sex, is uncertain.

**Hypothesis:**

Heavy alcohol consumption is associated with worse CVH.

**Methods:**

We explored associations between alcohol consumption and CVH in a multi‐ethnic population including 6506 participants free of CVD, aged 45 to 84 years. Each LS7 metric was scored 0 to 2 points. Total score was categorized as inadequate (0‐8), average (9‐10) and optimal (11‐14). Participants were classified as never, former or current drinkers. Current drinkers were categorized as <1 (light), 1 to 2 (moderate) and >2 (heavy) drinks/day. Multinomial logistic regression models assessed associations between alcohol and CVH, adjusted for age, sex, race/ethnicity, education, income, and health insurance.

**Results:**

Mean (SD) age was 62 (10) years, 53% were women. Compared to never drinkers, those with >2 drinks/day were less likely to have average [0.61 (0.43‐0.87)] and optimal CVH [0.29 (0.17‐0.49)]. Binge drinking was also associated with unfavorable CVH. Overall, there was no independent association for light or moderate drinking with CVH. However, women with 1 to 2 drinks/day were more likely to have optimal CVH [1.85 (1.19‐2.88)] compared to non‐drinking women, which was not seen in men.

**Conclusion:**

Heavy alcohol consumption was associated with unfavorable CVH. Although light or moderate drinking may be associated with a more favorable CVH in women, overall, the association was not strong.

## 1 INTRODUCTION

Globally, heavy alcohol consumption is responsible for considerable socioeconomic burden causing 3.3 million deaths in 2012.^1^, ^2^ In the United States, an estimated 62 000 men and 26 000 women die annually from alcohol‐related causes^1^, ^3^ which makes alcohol the third leading preventable cause of death, behind tobacco use, and poor dietary habits/physical inactivity.^1^, ^4^ Alcohol misuse in the United States cost approximately $250 billion in 2010 and 3/4 of this was attributable to binge drinking.^1^, ^5^ However, literature is replete with research suggesting an inverse relationship between light to moderate alcohol consumption and cardiovascular disease (CVD), as well as all‐cause mortality.^6^, ^7^ For example, a review of 84 studies reported that alcohol consumption of 1 drink or less per day was consistently associated with a 14% to 25% reduction in the risk of all the health outcomes evaluated, which included CVD, compared to those who abstained from alcohol.^7^ Although the effects of alcohol on CVD has been well researched and documented,^8^, ^9^ research exploring the association between alcohol consumption and intermediate measures of cardiovascular health (CVH), assessed using the American Heart Association's Life's Simple 7 (LS7) metrics,^10^, ^11^ is sparse.^12^ Even less is known about sex, age, and race/ethnic differences in these associations. The LS7 metrics were introduced to measure and monitor the CVH of individuals and populations where ideal CVH is defined as the presence of both ideal health behaviors (non‐smoking, body mass index [BMI] <25 kg/m^2^, physical activity at goal levels and diet consistent with guidelines) and ideal health factors (untreated total cholesterol ≤200 mg/dL, untreated blood pressure <120/mg and untreated fasting blood glucose <100 mg/dL).^10^, ^11^ We examined the cross‐sectional associations between alcohol consumption and ideal CVH using data from the Multi‐Ethnic Study of Atherosclerosis (MESA), a large multi‐center prospective cohort study. We hypothesized that heavy alcohol consumption will be associated with worse CVH independent of sociodemographic factors, which include age, sex, race/ethnicity, education, income, and health insurance status.

## 2 METHODS

A detailed methodology of MESA has been previously described.^13^ Briefly, the MESA study recruited 6814 adults between July 2000 and August 2002 from 6 U.S. centers (Baltimore, MD; Chicago, IL; Forsyth County, NC; Los Angeles, CA; New York, NY and St Paul, MN). The study included men and women aged 45 to 84 years, without a previous history of clinical CVD at baseline. Approximately 38% were White, 28% Black, 23% Hispanic, and 11% Chinese American. Study participants gave informed consent and the institutional review boards of each recruitment center approved the study protocol. Standardized questionnaires, physical exam, and fasting laboratory draw were used to obtain information from participants. We included 6506 participants from MESA baseline data after excluding those without complete information on the LS7 metrics (n = 308). Details of the assessments of the Life's Simple 7 metrics can be found in Appendix S1, Supporting Information.

Alcohol consumption was assessed from questions in a personal history questionnaire. Participants were asked the following questions: “Have you ever consumed alcoholic beverages?” If yes, they were also asked “Do you presently drink alcoholic beverages?” Answers given to these questions were used to classify participants into never, former, and current drinkers. Current and former drinkers were asked about the number of years of drinking, and the usual number of drinks consumed per week (before they stopped drinking in the case of former drinkers). In addition, current drinkers were asked about the number of drinks consumed in the past 24 hours and the largest number of drinks consumed in 1 day in the past month. These questions were used to know participants who engage in binge drinking, defined as the consumption of 5 or more drinks on 1 occasion in the past month.^13^, ^14^

Information was collected on sociodemographic variables, which included age, sex, race/ethnicity, educational attainment, income, and health insurance.^13^ In our stratified analysis, age, and sex had two categories, <65 and ≥65 years; men and women, respectively. Race/ethnicity had four categories, White, Chinese American, Black, and Hispanic. Education was classified as ≥bachelor's degree and < bachelor's degree. Income was divided into participants who made ≥$40 000 and < $40 000 while health insurance was grouped into “Yes” and “No.”

### 2.1 Statistical analysis

The characteristics of the study participants were reported by categories of alcohol consumption. Categorical variables were presented as frequencies with percentages, and continuous variables were presented as means with standard deviation (SD). Descriptive statistics were used to compare the baseline characteristics of all participants by alcohol consumption categories, using anova for continuous variables and *χ* ^2^ tests for categorical variables.

The LS7 metrics were each categorized into ideal, intermediate, and poor,^10^ as shown in Table S1. Points were awarded to each category with 0 indicating poor; 1, intermediate; and 2, ideal. The points were summed, yielding a total CVH score ranging from 0 to 14.^15^ As previously reported, CVH scores of 0 to 8, 9 to 10, and 11 to 14 were considered inadequate, average, and optimal, respectively.^16^, ^17^, ^18^, ^19^, ^20^, ^21^

We reported the proportions of the individual LS7 metrics by alcohol consumption categories. Multinomial logistic regression modeling was used to examine the association between alcohol consumption and CVH score. Two separate regression models were fitted. Model 1 was unadjusted and model 2 was adjusted for sociodemographic factors (age, sex, race/ethnicity, education, income, and health insurance status). Odds ratios (ORs) and their 95% confidence intervals (CIs) were calculated for average and optimal CVH score across the categories of alcohol consumption. Consistent with prior studies,^14^, ^16^, ^18^ the reference groups were the inadequate score for the CVH categories and the “never” categories for alcohol consumption and binge drinking.

We examined the interaction of alcohol consumption with sex, race/ethnicity, and age using the likelihood ratio *χ* ^2^ test, by including interaction terms in model 2. The association between alcohol consumption and CVH score was stratified by sex, race/ethnicity, and age. All analyses were performed using STATA version 14.1 (StataCorp LP, College Station, Texas) and a two‐sided *P*‐value <0.05 was considered statistically significant.

## 3 RESULTS

The baseline characteristics of the 6506 study participants varied across alcohol consumption categories as reported in Table 1. Fifty‐three percent of participants were women, and the mean age (SD) was 62 (10) years. Among participants, 20% were never drinkers, 24% were former drinkers and 56% were current drinkers. Of the current drinkers, 78% consumed <1 drink/day (mild); 16%, 1 to 2 drinks/day (moderate) and 6%, >2 drinks/day (heavy). In addition, 14% of current drinkers reported binge drinking in the past month. Of note, healthy diet score decreased as alcohol consumption increased. For the overall cohort, 47%, 33%, and 20% met criteria for inadequate, average and optimal CVH scores, respectively. The proportion of participants with optimal CVH scores were lowest in the >2 drinks/day and binge drinking categories.

**Table 1:** Characteristics of study participants by alcohol consumption: MESA (N = 6506)

Alcohol consumption								
	Total (N = 6506)	Never (n = 1322)	Former (n = 1533)	<1 drink/day (n = 2855)	1‐2 drinks/day (n = 579)	>2 drinks/day (n = 217)	Binge (n = 524)	*P*‐value*
Age, mean (SD), years	62 (10)	64 (10)	63 (10)	61 (10)	62 (10)	59 (10)	58 (9)	<0.0001
<65 years	3713 (57%)	665 (50%)	817 (53%)	1761 (62%)	324 (56%)	146 (67%)	390 (74%)	<0.0001
≥65 years	2793 (43%)	657 (50%)	716 (47%)	1094 (38%)	255 (44%)	71 (33%)	134 (26%)	
Sex								
Men, n (%)	3074 (47%)	312 (24%)	806 (53%)	1385 (49%)	378 (65%)	193 (89%)	430 (82%)	<0.0001
Women, n (%)	3432 (53%)	1010 (76%)	727 (47%)	1470 (51%)	201 (35%)	24 (11%)	94 (18%)	
Race/ethnicity								
White	2539 (39%)	232 (18%)	474 (31%)	1339 (47%)	349 (60%)	145 (67%)	239 (46%)	<0.0001
Chinese American	795 (12%)	426 (32%)	119 (8%)	223 (8%)	22 (4%)	5 (2%)	11 (2%)	
Black	1716 (26%)	287 (22%)	552 (36%)	727 (25%)	120 (21%)	30 (14%)	105 (20%)	
Hispanic	1456 (22%)	377 (29%)	388 (25%)	566 (20%)	88 (15%)	37 (17%)	169 (32%)	
Education								
≥Bachelor's degree	2331 (36%)	311 (24%)	415 (27%)	1232 (43%)	283 (49%)	90 (41%)	162 (31%)	<0.0001
<Bachelor's degree	4175 (64%)	1011 (76%)	1118 (73%)	1623 (57%)	296 (51%)	127 (59%)	362 (69%)	
Income								
≥$40 000	3214 (49%)	407 (31%)	587 (38%)	1701 (60%)	369 (64%)	150 (69%)	303 (58%)	<0.0001
<$40 000	3292 (51%)	915 (69%)	946 (62%)	1154 (40%)	210 (36%)	67 (31%)	221 (42%)	
Health insurance								
Yes	5925 (91%)	1126 (85%)	1395 (91%)	2655 (93%)	548 (95%)	201 (93%)	477 (91%)	<0.0001
No	581 (9%)	196 (15%)	138 (9%)	200 (7%)	31 (5%)	16 (7%)	47 (9%)	
LS7 metrics								
Current smoking	839 (13%)	80 (6%)	187 (12%)	391 (14%)	112 (19%)	69 (32%)	173 (33%)	<0.0001
Body mass index kg/m^2^	28 (6)	28 (6)	29 (6)	28 (5)	27 (5)	28 (4)	29 (4)	<0.0001
Physical activity MET‐min/week	402 (605)	320 (539)	372 (594)	442 (630)	459 (606)	418 (674)	451 (737)	<0.0001
Healthy diet score (0–5)	1.6 (0.9)	1.7 (0.9)	1.5 (0.9)	1.5 (0.9)	1.5 (0.9)	1.3 (0.9)	1.2 (0.8)	<0.0001
Total cholesterol mg/dL	194 (36)	197 (36)	190 (36)	195 (36)	195 (35)	201 (35)	198 (38)	<0.0001
Systolic blood pressure mm Hg	126 (21)	129 (22)	128 (22)	124 (21)	126 (21)	126 (18)	126 (20)	<0.0001
Diastolic blood pressure mmHg	72 (10)	71 (10)	72 (10)	72 (10)	73 (10)	76 (10)	76 (10)	<0.0001
Fasting blood glucose mg/dl	97 (30)	100 (33)	100 (34)	95 (27)	95 (27)	98 (28)	98 (30)	<0.0001
Categories of ideal LS7 metrics								
0‐2	1710 (26%)	358 (27%)	479 (31%)	652 (23%)	138 (24%)	83 (38%)	190 (36%)	<0.0001
3‐5	4526 (70%)	923 (70%)	991 (65%)	2072 (73%)	411 (71%)	129 (59%)	326 (62%)	
6‐7	270 (4%)	41 (3%)	63 (4%)	131 (5%)	30 (5%)	5 (2%)	8 (2%)	
CVH score, n (%)								
Inadequate (0‐8)	3080 (47%)	630 (48%)	828 (54%)	1230 (43%)	259 (45%)	133 (61%)	327 (62%)	
Average (9, 10)	2120 (33%)	433 (33%)	448 (22%)	992 (35%)	183 (32%)	64 (29%)	144 (27%)	<0.0001
Optimal (11‐14)	1306 (20%)	259 (20%)	257 (17%)	633 (22%)	137 (24%)	20 (9%)	53 (10%)	

Abbreviations: CVH, Cardiovascular health; MESA, Multi‐Ethnic Study of Atherosclerosis; SD, Standard deviation; LS7, Life's Simple 7’.

Percentages/numbers were rounded up to whole numbers except diet; LS7 metrics are expressed in mean (SD) except smoking; *P‐values compare differences between never, former, <1, 1 to 2, and >2; Total does not include binge drinking.

The distribution of the individual LS7 metrics by alcohol consumption in the study population is reported in Table 2 . With the exception of physical activity and blood glucose, the proportion of never drinkers who met the ideal criteria for the LS7 metrics was larger than that for current drinkers who consumed more than 2 drinks/day. None of the participants who reported more than 2 drinks/day or binge drinking met the ideal criteria for diet.

**Table 2:** Distribution of Life's Simple 7 metrics by alcohol consumption

Alcohol consumption								
	Total	Never	Former	<1 drink/day	1–2 drinks/day	>2 drinks/day	Binge	P*
Smoking								
Poor	839 (13%)	80 (6%)	187 (12%)	391 (14%)	112 (19%)	69 (32%)	173 (33%)	<0.0001
Intermediate	80 (1%)	6 (1%)	25 (2%)	38 (1%)	5 (1%)	6 (3%)	15 (3%)	
Ideal	5587 (86%)	1236 (93%)	1321 (86%)	2426 (85%)	462 (8%)	142 (65%)	336 (64%)	
Body mass index								
Poor	2073 (32%)	380 (29%)	600 (39%)	889 (31%)	143 (25%)	61 (28%)	180 (34%)	<0.0001
Intermediate	2558 (39%)	489 (37%)	567 (37%)	1162 (41%)	238 (41%)	102 (47%)	235 (45%)	
Ideal	1875 (29%)	452 (34%)	366 (24%)	804 (43%)	198 (34%)	54 (25%)	109 (21%)	
Physical activity								
Poor	1486 (23%)	399 (30%)	411 (27%)	518 (18%)	102 (18%)	56 (26%)	132 (25%)	<0.0001
Intermediate	1128 (17%)	228 (17%)	263 (17%)	513 (18%)	85 (15%)	39 (18%)	73 (14%)	
Ideal	3892 (60%)	695 (53%)	859 (56%)	1824 (64%)	392 (68%)	122 (56%)	319 (61%)	
Diet								
Poor	2943 (45%)	467 (35%)	721 (47%)	1342 (47%)	284 (49%)	129 (59%)	325 (62%)	<0.0001
Intermediate	3493 (54%)	833 (63%)	802 (52%)	1483 (52%)	287 (50%)	88 (41%)	199 (38%)	
Ideal	70 (1%)	22 (2%)	10 (1%)	30 (1%)	8 (1%)	0 (0%)	0 (0%)	
Total cholesterol								
Poor	872 (18%)	192 (15%)	168 (11%)	402 (14%)	72 (12%)	38 (18%)	88 (17%)	0.015
Intermediate	2544 (39%)	518 (39%)	598 (39%)	1094 (38%)	246 (43%)	88 (41%)	204 (39%)	
Ideal	3090 (47%)	612 (46%)	767 (50%)	1359 (48%)	261 (45%)	91 (42%)	232 (44%)	
Blood pressure								
Poor	2439 (37%)	586 (44%)	605 (40%)	961 (34%)	209 (36%)	78 (36%)	172 (33%)	<0.0001
Intermediate	1819 (28%)	328 (25%)	461 (30%)	789 (28%)	165 (29%)	76 (35%)	173 (33%)	
Ideal	2248 (35%)	408 (31%)	467 (30%)	1105 (39%)	205 (35%)	63 (29%)	179 (34%)	
Blood glucose								
Poor	700 (11%)	190 (14%)	223 (15%)	236 (8%)	36 (6%)	15 (7%)	52 (10%)	<0.0001
Intermediate	987 (15%)	200 (15%)	256 (17%)	393 (14%)	94 (16%)	44 (20%)	87 (17%)	
Ideal	4819 (74%)	932 (71%)	1054 (69%)	2226 (78%)	449 (78%)	158 (73%)	385 (73%)	

Percentages were rounded up to whole numbers; *P‐value compares differences between never, former, <1, 1–2, and >2; Total does not include binge drinking.

The associations between alcohol consumption and CVH in the overall cohort and stratified by sex, age, and race/ethnicity are reported in Table 3 , Table S2 and S3, respectively. Compared to never drinkers, participants in the overall cohort who were heavy drinkers (>2 drinks/day) were less likely to have average [0.61 (0.43‐0.87) and optimal [0.29 (0.17‐0.49)] CVH scores in the adjusted analysis. In addition, participants who reported binge drinking in the past month were 34% and 61% less likely to have average and optimal CVH **(**Table 3 **).** In sex‐stratified results (Table 3), men who drank >2 drinks/day were 76% less likely to have optimal scores. Binge drinking was associated with unfavorable CVH for both men and women. In age‐stratified results (Table S2), participants who drank >2 drinks/day had lower odds of having optimal CVH for both those <65 years [0.31 (0.16‐0.59)] and for those ≥65 years [0.31 (0.12‐0.78)], compared to non‐drinkers within their respective age groups. In results stratified by race/ethnicity (Table S3), White participants who drank >2 drinks/day had lower odds of optimal scores [0.31 (0.16‐0.60)]; binge drinking was unfavorable for CVH for Whites, Hispanics, and Blacks. Evaluation of the associations of heavy and binge drinking with CVH for the other race/ethnic groups were limited by sample size. In summary, there were fairly consistent findings of an inverse relationship of heavy and binge drinking with ideal CVH that was noted across age, sex, and race/ethnic groups.

**Table 3:** Multivariable association between alcohol consumption and cardiovascular health, by overall cohort and sex

	Overall cohort		Women		Men	
	Average vs inadequate	Optimal vs inadequate	Average vs inadequate	Optimal vs inadequate	Average vs inadequate	Optimal vs inadequate
	OR (95% CI)		OR (95% CI)		OR (95% CI)	
Model 1: unadjusted						
Usual consumption						
Never	1 [reference]	1 [reference]	1 [reference]	1 [reference]	1 [reference]	1 [reference]
Former	*0.79 (0.67‐0.93)*	*0.75 (0.62‐0.92)*	*0.68 (0.54‐0.84)*	*0.63 (0.48‐0.83)*	0.82 (0.60‐1.10)	*0.70 (0.50‐0.99)*
<1 drink/day	*1.17 (1.01‐1.36)*	*1.25 (1.05‐1.49)*	*1.32 (1.10‐1.59)*	*1.51 (1.22‐1.88)*	0.94 (0.71‐1.25)	0.84 (0.61‐1.15)
1‐2 drinks/day	1.03 (0.82‐1.29)	1.29 (1.00‐1.65)	*1.47 (1.02‐2.12)*	*2.64 (1.82‐3.86)*	0.78 (0.55‐1.09)	*0.66 (0.44‐0.98)*
>2 drinks/day	*0.70 (0.51‐0.97)*	*0.37 (0.22‐0.60)*	0.40 (0.13‐1.23)	0.90 (0.32‐2.50)	*0.64 (0.43‐0.96)*	*0.23 (0.12‐0.42)*
Binge drinking past month						
No (never)	1 [reference]	1 [reference]	1 [reference]	1 [reference]	1 [reference]	1 [reference]
No (current)	*1.23 (1.06‐1.42)*	*1.38 (1.17‐1.64)*	*1.39 (1.16‐1.66)*	*1.73 (1.40‐2.13)*	0.99 (0.75‐1.31)	0.90 (0.66‐1.23)
Yes	*0.64 (0.51‐0.81)*	*0.39 (0.29‐0.55)*	*0.61 (0.37‐0.99)*	*0.45 (0.22‐0.89)*	*0.57 (0.41‐0.80)*	*0.30 (0.19‐0.44)*
*Model 2: adjusted						
Usual consumption						
Never	1 [reference]	1 [reference]	1 [reference]	1 [reference]	1 [reference]	1 [reference]
Former	0.95 (0.79‐1.15)	1.04 (0.83‐1.31)	0.84 (0.67‐1.06)	0.83 (0.60‐1.13)	1.06 (0.77‐1.46)	1.15 (0.79‐1.66)
<1 drink/day	1.16 (0.98‐1.37)	1.17 (0.93‐1.44)	*1.33 (1.07‐1.64)*	1.27 (0.97‐1.66)	1.01 (0.75‐1.36)	1.00 (0.70‐1.41)
1‐2 drinks/day	0.98 (0.76‐1.25)	1.15 (0.86‐1.54)	1.32 (0.89‐1.97)	*1.85 (1.19‐2.88)*	0.82 (0.57‐1.18)	0.78 (0.51‐1.20)
>2 drinks/day	*0.61 (0.43‐0.87)*	*0.29 (0.17‐0.49)*	0.34 (0.11‐1.07)	0.50 (0.16‐1.52)	*0.63 (0.41‐0.96)*	*0.24 (0.13‐0.46)*
Binge drinking past month						
No (never)	1 [reference]	1 [reference]	1 [reference]	1 [reference]	1 [reference]	1 [reference]
No (current)	*1.19 (1.01‐1.41)*	*1.25 (1.02‐1.53)*	*1.37 (1.11‐1.69)*	*1.39 (1.07‐1.82)*	1.03 (0.76‐1.38)	1.02 (0.73‐1.43)
Yes	*0.66 (0.51‐0.85)*	*0.39 (0.27‐0.56)*	0.61 (0.36‐1.03)	*0.33 (0.16‐0.70)*	*0.64 (0.45‐0.92)*	*0.38 (0.24‐0.61)*

Abbreviation: OR, odds ratio.

*Adjusted for socio‐demographic factors: age, sex, race/ethnicity, education, income and health insurance status; OR < 1 is interpreted as decreased odds of having an optimal or average cardiovascular health score. Results in italicized font are statistically significant; *P* < 0.05.

Regarding light or moderate drinking, in unadjusted analysis, light drinking (<1 drink/day) was associated with greater odds of optimal CVH [1.25 (1.05‐1.49)] for the overall cohort; however, this was attenuated and no longer statistically significant after multivariable adjustment [1.17 (0.93‐1.44)]. Yet in sex‐stratified analyses, women who drank 1 to 2 drinks/day were 85% more likely to have optimal CVH compared to non‐drinking women [1.85 (1.19‐2.88)], while no similar association was seen for men.

Although results were stratified given a priori interest in the relation of alcohol consumption and CVH among subgroups, we formally tested for interaction of the associations by sex, age, and race/ethnicity. For the CVH scores, there was a significant interaction for alcohol consumption with sex (usual consumption: *P* = 0.009 for average scores and < 0.001 for optimal scores; Binge drinking: *P* = 0.024 for optimal scores). However, the interaction between alcohol consumption and race/ethnicity or age was not statistically significant.

**Figure 1:**
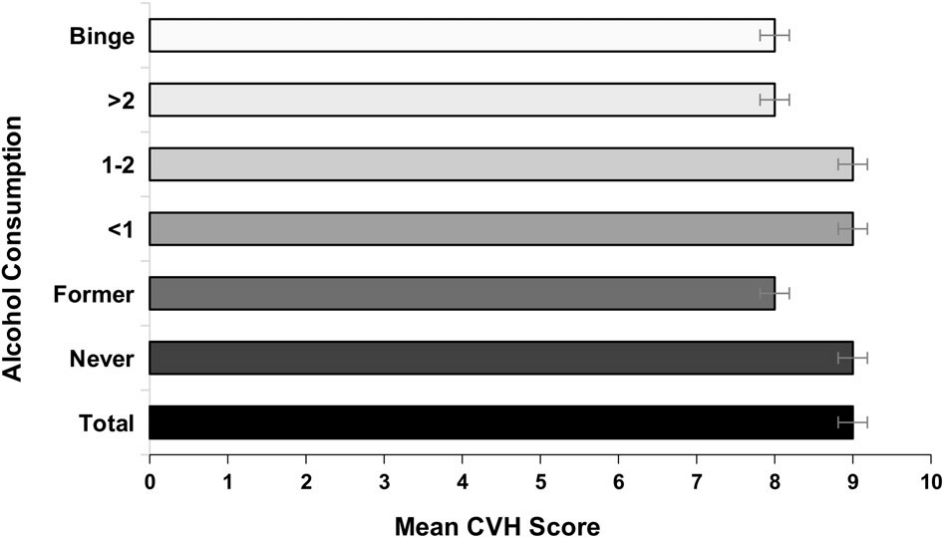
Mean CVH score (and SE) for each alcohol intake category. First bar: Total alcohol consumption; second bar: Never drinkers; third bar: Former drinkers; fourth bar: <1drink/day; fifth bar: 1 to 2 drinks/day; sixth bar: >2 drinks/day; seventh bar: Binge drinking in the past month. CVH score ranges from 0 to 14. CVH, cardiovascular health

The distribution of the mean CVH score by alcohol consumption categories are shown in Figure 1. Mean CVH score was slightly lower for former drinkers and participants who consumed more than 2 drinks/day as well as for those who reported binge drinking (Figure 1). The associations of alcohol consumption with each LS7 metric are shown in Table S4. Regardless of quantity of alcohol consumed, participants had lower odds of achieving the ideal criteria for smoking. Participants with <1 and 1 to 2 drinks/day had higher odds of achieving the ideal criteria for physical activity, BMI, and blood glucose. For total cholesterol, those who reported >2 drinks/day were 54% less likely to meet the ideal criteria, while for blood pressure, those who reported light intake (<1 drink/day) were 38% more likely to meet the ideal criteria.

## 4 DISCUSSION

### 4.1 Main findings

In this multi‐ethnic cohort study of adults free of CVD at baseline, we found that after adjusting for sociodemographic characteristics, most study participants who reported <1 drink/day (light) and 1 to 2 drinks/day (moderate) were more likely to have average and optimal CVH scores, but the association was only statistically significant for women. In contrast, in the overall cohort and in the stratified analyses by sex, age, and race/ethnicity, participants who consumed more than 2 drinks/day (heavy) or reported binge drinking in the past month had lower odds of having average and optimal CVH scores compared to never drinkers or those who did not report binge drinking, respectively. However, because of the cross‐sectional design of this study, we cannot prove a causal relationship between alcohol consumption and CVH.

### 4.2 Comparison to previous studies

Previous studies have examined the association between alcohol and CVD.^6^, ^7^, ^22^ For example, a 2018 review conducted by O'Keefe et al. titled “Alcohol and CV health: Jekyll and Hyde J‐Curves,” reported excessive alcohol consumption is a common cause of reversible hypertension, non‐ischemic dilated cardiomyopathy, atrial fibrillation, and stroke. The authors also reported that though light to moderate alcohol consumption (≤1 drink/day for women and 1‐2 drinks/day for men) was associated with lower risk for all‐cause mortality, coronary heart disease, type 2 diabetes, heart failure, and stroke, the risk‐benefit ratio of drinking may be less favorable in younger individuals, because they are more likely to abuse alcohol which is a leading cause of premature death among people between 15 and 59 years.^22^

### 4.3 Possible mechanisms

The main finding of our study showed light, and in some participants moderate, alcohol consumption was associated with favorable CVH scores and most ideal metrics. Participants who drank any alcohol did not meet the ideal criteria for smoking. This is not surprising because alcohol consumption and smoking are closely related behaviors.^23^ Among alcoholics, the rate of smoking is approximately 90% and may be attributable to neurobiological mechanisms, such as cross‐tolerance and cross‐sensitization to both nicotine and alcohol.^23^ Light to moderate alcohol consumption was associated with increased odds of ideal physical activity and BMI. Although these positive associations have been previously reported,^24^, ^25^, ^26^ the mechanisms underlying them are still ambiguous. For example, individuals who drink alcohol only in moderation may have other favorable health‐seeking behaviors that contribute to their more optimal CVH profile.

In addition, we found light to moderate alcohol consumption was associated with ideal blood glucose levels. This finding, if causal, could be explained by the hypoglycemic effect alcohol has on glucose metabolism. Light to moderate alcohol consumption has been linked to increase in high‐density lipoprotein (HDL) cholesterol; however, we found that participants who reported >2 drinks/day were 54% less likely to have ideal total cholesterol levels. For blood pressure, those who reported <1 drink/day were 38% more likely to have ideal levels. Prior research has demonstrated heavy drinking increases blood pressure mainly through stimulation of the sympathetic nervous system and increase in intracellular calcium in the vascular smooth muscle.^27^, ^28^, ^29^

Another interesting finding of our study was that sex was an effect modifier of the association between alcohol and CVH. Similar to prior research,^30^ we found that women had a larger proportion of abstainers compared to men (29% vs 10%) while men had a larger proportion of heavy (6% vs <1%) and binge drinkers (14% vs 3%). These differences in drinking habits may be driven by psycho‐socio‐cultural factors, such as the perceived differences in traditional gender roles observed in different ethnic and age groups.^30^, ^31^, ^32^, ^33^, ^34^ However, recent studies show that the differences reported in alcohol consumption among men and women is diminishing as the socioeconomic and cultural characteristics of younger people become more similar regardless of sex.^30^, ^31^, ^32^, ^33^, ^34^

### 4.4 Implications

Despite the fact that our results showed light and some moderate drinkers were more likely to have better CVH, we confirmed that heavy and binge drinkers were more likely to have poor CVH. Thus, it would still be inappropriate to recommend that everyone should consume 1 to 2 drinks of alcohol per day because of the high‐risk for addiction. Prior research has shown that approximately half of all drinkers have at least short‐term problems with alcohol and an estimated 10% of all drinkers are alcoholics.^35^, ^36^ Alcohol consumption also plays a role in the incidence of over 200 diseases and injury‐related health conditions accounting for 5.1% of the burden of disease and injury worldwide.^1^, ^2^In addition, a recently published paper that examined the risk threshold for alcohol consumption recommended lower limits for alcohol consumption.^37^ The results of the study showed that in high‐income countries, such as the United States, the threshold for the lowest risk for all‐cause mortality was about 100 g/week (approximately 1 drink/day) in contrast to threshold limits in the current United States guidelines of 196 g/week for men and 98 g/week for women. The risk for stroke, coronary disease (excluding myocardial infarction), heart failure, fatal hypertensive heart disease, and fatal aortic aneurysm was 14%, 6%, 9% 24%, and 15% higher per 100 g per week higher consumption, respectively. However, a 6% lower risk was reported for myocardial infarction. Furthermore, people who reported drinking >100 to ≤200 g per week, >200 to ≤350 g per week, or > 350 g per week had a lower life expectancy compared to those who reported 0 to 100 g per week.^37^ This is consistent with our findings of an association with a favorable CVH profile for light drinking but an unfavorable CVH profile for heavy or binge drinking.

### 4.5 Strengths and limitations

Our study has many strengths including the large, ethnically diverse population that allowed for stratification by sex, age, and race/ethnicity. In addition, data collection for the measurements of CVH and alcohol were performed using standardized methods and procedures. This study also has limitations. First, the use of self‐report questionnaires may have introduced recall bias for the collection of data on alcohol, smoking, physical activity, and diet. Second, because some participants may have given socially acceptable responses to questions about alcohol consumption, there is a possibility that alcohol consumption may have been under‐reported which may lead to non‐differential misclassification and bias the associations toward the null. Third, this study is observational, and although we adjusted for confounding factors such as demographics and socioeconomic status, there may be residual confounding factors that distinguish people who chose to drink vs not. Fourth, the study is cross‐sectional so we are unable to determine temporality of the associations seen. Fifth, many of the associations examined may not have been statistically significant because of multiple comparisons. We were unable to make inferences regarding ethnic differences for Chinese Americans and Blacks with optimal CVH who reported >2 drinks/day and for Chinese Americans who reported binge drinking due to the relatively small sample sizes in these categories.

## 5 CONCLUSION

In summary, using the relatively new health metric of the LS7 criteria,^10^ this study explores the relationship between alcohol and CVH, which represents a paradigm shift from a focus on CVD prevention to promotion of CVH and wellness.^11^ Given that alcohol consumption is common in developed countries, our study was designed to further the understanding of the relationship of various levels of alcohol consumption with CV health and behavioral factors. We showed that light alcohol consumption was associated with favorable CVH, whereas heavy alcohol consumption was unfavorable. However, we do not recommend that abstainers start drinking because alcohol has a high addiction potential and several adverse health consequences. Current drinkers should limit consumption to ≤100 g per week in line with recently published research that shows this threshold to be associated with a lower risk of CVD and all‐cause mortality.^37^ Future research could investigate the association between alcohol consumption and CVH in a younger cohort because they consume a higher amount of alcohol and binge drinking is more commonly reported among them.^22^ As suggested in prior research,^8^ biomarkers of alcohol, such as PEth can be employed to corroborate self‐reported use of alcohol and differentiate between light, moderate, and heavy consumption.^8^ In addition, the role sex plays as an effect modifier of the association between alcohol consumption and CVH could be further examined in future studies.

## CONFLICTS OF INTEREST

The authors declare no potential conflict of interests.

## Supporting information


**Appendix S1** Methodology: assessment of Life's Simple 7 metrics
**Table S1** Distribution of Life's Simple 7 metrics
**Table S2** Multivariable association between alcohol consumption and cardiovascular health, by overall cohort and age
**Table S3** Multivariable association between alcohol consumption and cardiovascular health, by race/ethnicity
**Table S4** Multivariable association between alcohol consumption and Life's simple 7 metrics
**Table S5** Multivariable linear regression of the association between alcohol consumption and cardiovascular health
