# Supplementary material for: Alcohol and ideal cardiovascular health: The Multi‐Ethnic Study of Atherosclerosis
Source: Clin Cardiol. 2018 Dec 17;42(1):151–8. doi: 10.1002/clc.23125 (PMC6357768; doi:10.1002/clc.23125)
Supplement: Supplementary file 1 — Appendix S1 Methodology: assessment of Life's Simple 7 metrics Table S1 Distribution of Life's Simple 7 metrics Table S2 Multivariable association between alcohol consumption and cardiovascular health, by overall cohort and age Table S3 Multivariable association between alcohol consumption and cardiovascular health, by race/ethnicity Table S4 Multivariable association between alcohol consumption and Life's simple 7 metrics Table S5 Multivariable linear regression of the association between alcohol consumption and cardiovascular health [file CLC-42-151-s001.docx]

**SUPPLEMENTAL MATERIAL**

Methodology: Assessment of Life’s Simple 7 Metrics

Supplemental Table 1. Distribution of Life’s Simple 7 Metrics

Supplemental Table 2 Multivariable Association between Alcohol Consumption and Cardiovascular Health, by Overall Cohort and Age

Supplemental Table 3. Multivariable Association between Alcohol Consumption and Cardiovascular Health, by Race/Ethnicity

Supplemental Table 4. Multivariable Association between Alcohol Consumption and Life’s Simple 7 Metrics

Supplemental Table 5. Multivariable Linear Regression of the Association between Alcohol Consumption and Cardiovascular Health

**Methodology**

*Assessment of Life’s Simple 7 Metrics*

Smoking status was assessed from self-reports of participants, and classified as current, former (quit within the last 12 months) and nonsmokers (never smoked or quit more than 12 months ago) [1]. In assessing physical activity, participants responded to a self-report survey instrument adapted from the Cross-Cultural Activity Participation Study [2] containing questions on time and frequency of activities during a week in the previous month. The survey has 28 questions including; conditioning activities, care of children/adults, dancing and sports activities, household chores, lawn/yard/garden/farm activities, leisure activities, occupational activities and volunteer activities. We included as exercise, minutes spent during activities such as conditioning, leisure, and walking, and used them in the calculation of the total minutes of moderate and vigorous exercise [3].

Body mass index was calculated using the measurements of weight and height expressed in kg/m^2^. For dietary assessment, a 120-item validated food frequency questionnaire modified from the Insulin Resistance Atherosclerosis Study instrument [4, 5] was utilized. The 5 components of the healthy diet were defined by the AHA and comprised fruits and vegetables, fish, whole grains and intake of sodium <1500mg per day and sugar-sweetened beverages ≤450 kcal (36 oz.) per week [1]. Three blood pressure measurements were obtained from each participant in a seated position after a 5 minutes rest and the average value for the last 2 readings were recorded as the blood pressure metric. Total cholesterol and blood glucose levels were measured from blood samples after a 12-hour fast.

| **Supplemental Table 1. Distribution of Life’s Simple 7 Metrics** | | | |
| --- | --- | --- | --- |
| **LS7 Metrics** | **Score** | **Definition** | **% MESA Participants, N=6506** |
|  |  |  |  |
| **Smoking** | 0 | Current smoker | 12.9% |
|  | 1 | Former smoker, quit ≤12 mo ago | 1.2% |
|  | 2 | Never smoker or quit >12 mo ago | 85.9% |
| **Body Mass Index** | 0 | ≥30 kg/m^2^ | 31.9% |
|  | 1 | 25.0–29.99 kg/m^2^ | 39.3% |
|  | 2 | <25.0 kg/m^2^ | 28.8% |
| **Physical Activity*** | 0 | No exercise | 22.8% |
|  | 1 | 1–149 min of moderate exercise or 1–74 min of vigorous exercise/week | 17.3% |
|  | 2 | 150+ min of moderate exercise or 75+ min of vigorous exercise/week | 59.8% |
| **Diet** | 0 | 0–1 components of healthy diet | 45.2% |
|  | 1 | 2–3 components of healthy diet | 53.7% |
|  | 2 | 4–5 components of healthy diet | 1.1% |
| **Total Cholesterol** | 0 | ≥240 mg/dL | 13.4% |
|  | 1 | 200–239 mg/dL or treated to <200 mg/dL | 39.1% |
|  | 2 | <200 mg/dL, unmedicated | 47.5% |
| **Blood Pressure** | 0 | SBP ≥140 mmHg or DBP ≥90 mmHg | 37.5% |
|  | 1 | SBP 120–139 mmHg or DBP 80–89 mmHg or treated to <120/80 mmHg | 28.0% |
|  | 2 | <120/80 mm Hg, unmedicated | 34.6% |
| **Blood Glucose** | 0 | ≥126 mg/dL fasting | 10.8% |
|  | 1 | 100–125 mg/dL fasting or treated to <100 mg/dL | 15.2% |
|  | 2 | <100 mg/dL fasting, unmedicated | 74.1% |
| Adapted from Lloyd Jones et al [1] and Unger et al [3], DBP indicates diastolic blood pressure and SBP, systolic blood pressure. Poor=0 point, Intermediate=1 point, ideal =2 points. *When combining vigorous and moderate exercise, vigorous exercise was weighted double; mo, month | | | |

| **Supplemental Table 2 Multivariable Association Between Alcohol Consumption and Cardiovascular Health, by Overall Cohort and Age** | | | | | | |
| --- | --- | --- | --- | --- | --- | --- |
|  | Overall Cohort | | <65 | | ≥65 | |
|  | Average vs. Inadequate | Optimal vs. Inadequate | Average vs. Inadequate | Optimal vs. Inadequate | Average vs. Inadequate | Optimal vs. Inadequate |
|  | OR (95% CI) | | OR (95% CI) | | OR (95% CI) | |
| ***Model 1: Unadjusted*** |  | | | | | |
| **Usual Consumption** | | | | | | |
| Never | 1 [Reference] | 1 [Reference] | 1 [Reference] | 1 [Reference] | 1 [Reference] | 1 [Reference] |
| Former | *0.79 (0.67-0.93)* | *0.75 (0.62-0.92)* | 0.86 (0.68-1.09) | 0.83 (0.63-1.08) | *0.71 (0.56-0.90)* | *0.65 (0.48-0.89)* |
| <1 drink/day | *1.17 (1.01-1.36)* | *1.25 (1.05-1.49)* | 1.13 (0.92-1.39) | 1.15 (0.91-1.44) | 1.21 (0.97-1.50) | 1.30 (1.00-1.70) |
| 1-2 drinks/day | 1.03 (0.82-1.29) | 1.29 (1.00-1.65) | 0.94 (0.69-1.27) | 1.02 (0.73-1.44) | 1.14 (0.82-1.60) | *1.67 (1.14-2.44)* |
| >2 drinks/day | *0.70 (0.51-0.97)* | *0.37 (0.22-0.60)* | *0.58 (0.39-0.88)* | *0.31 (0.17-0.56)* | 0.95 (0.56-1.61) | 0.44 (0.18-1.08) |
| **Binge drinking past month** | | | | | | |
| No (Never) | 1 [Reference] | 1 [Reference] | 1 [Reference] | 1 [Reference] | 1 [Reference] | 1 [Reference] |
| No (Current) | *1.23 (1.06-1.42)* | *1.38 (1.17-1.64)* | 1.20 (0.98-1.47) | *1.28 (1.01-1.60)* | *1.25 (1.01-1.55)* | *1.46 (1.12-1.89)* |
| Yes | *0.64 (0.51-0.81)* | *0.39 (0.29-0.55)* | 0.60 (0.45-0.80) | *0.36 (0.25-0.53)* | 0.72 (0.48-1.09) | *0.35 (0.18-0.70)* |
| ****Model 2: Adjusted*** |  | | | | | |
| **Usual Consumption** | | | | | | |
| Never | 1 [Reference] | 1 [Reference] | 1 [Reference] | 1 [Reference] | 1 [Reference] | 1 [Reference] |
| Former | 0.95 (0.79-1.15) | 1.04 (0.83-1.31) | 1.07 (0.82-1.38) | 1.19 (0.88-1.61) | 0.86 (0.66-1.11) | 0.90 (0.63-1.27) |
| <1 drink/day | 1.16 (0.98-1.37) | 1.17 (0.93-1.44) | 1.16 (0.92-1.46) | 1.20 (0.92-1.57) | 1.19 (0.93-1.52) | 1.15 (0.84-1.58) |
| 1-2 drinks/day | 0.98 (0.76-1.25) | 1.15 (0.86-1.54) | 0.95 (0.67-1.33) | 1.06 (0.72-1.56) | 1.03 (0.71-1.48) | 1.24 (0.80-1.92) |
| >2 drinks/day | *0.61 (0.43-0.87)* | *0.29 (0.17-0.49)* | *0.56 (0.36-0.88)* | *0.31 (0.16-0.59)* | 0.85 (0.48-1.51) | *0.31 (0.12-0.78)* |
| **Binge drinking past month** | | | | | | |
| No (Never) | 1 [Reference] | 1 [Reference] | 1 [Reference] | 1 [Reference] | 1 [Reference] | 1 [Reference] |
| No (Current) | *1.19 (1.01-1.41)* | *1.25 (1.02-1.53)* | 1.21 (0.96-1.53) | 1.28 (0.98-1.68) | 1.20 (0.94-1.52) | 1.21 (0.89-1.66) |
| Yes | *0.66 (0.51-0.85)* | *0.39 (0.27-0.56)* | *0.66 (0.48-0.91)* | *0.46 (0.30-0.70)* | 0.77 (0.49-1.21) | *0.34 (0.16-0.71)* |
| Abbreviation: OR indicates odds ratio. *Adjusted for socio-demographic factors: age, sex, race/ethnicity, education, income and health insurance status; OR <1 is interpreted as decreased odds of having an optimal or average cardiovascular health score. Results in italicized font are statistically significant; p<0.05. | | | | | | |

| **Supplemental Table 3. Multivariable Association Between Alcohol Consumption and Cardiovascular Health, by Race/Ethnicity** | | | | | | | | | |
| --- | --- | --- | --- | --- | --- | --- | --- | --- | --- |
|  | White | | Chinese American | | Black | | Hispanic | | |
|  | Average vs. Inadequate | Optimal vs. Inadequate | Average vs. Inadequate | Optimal vs. Inadequate | Average vs. Inadequate | Optimal vs. Inadequate | Average vs. Inadequate | Optimal vs. Inadequate | |
|  | OR (95% CI) | | OR (95% CI) | | OR (95% CI) | | OR (95% CI) | | |
| ***Model 1: Unadjusted*** |  | | | | | | | | |
| **Usual Consumption** | | | | | | | | | |
| Never | 1 [Reference] | 1 [Reference] | 1 [Reference] | 1 [Reference] | 1 [Reference] | 1 [Reference] | 1 [Reference] | | 1 [Reference] |
| Former | 0.76 (0.53-1.08) | 0.77 (0.51-1.17) | 1.17 (0.68-2.02) | *1.88 (1.10-3.23)* | 1.09 (0.79-1.52) | 1.30 (0.80-2.13) | 1.06 (0.76-1.47) | | 0.94 (0.61-1.45) |
| <1 drink/day | 1.30 (0.94-1.79) | *1.55 (1.08-2.23)* | 1.09 (0.73-1.63) | 1.21 (0.79-1.86) | 1.27 (0.93-1.74) | *1.71 (1.07-2.72)* | *1.43 (1.06-1.92)* | | 1.19 (0.80-1.76) |
| 1-2 drinks/day | 1.39 (0.94-2.05) | *1.85 (1.21-2.84)* | 0.37 (0.13-1.03) | 0.42 (0.14-1.24) | 0.91 (0.55-1.51) | 1.09 (0.52-2.26) | 0.74 (0.42-1.31) | | 0.84 (0.41-1.71) |
| >2 drinks/day | 0.77 (0.48-1.21) | *0.41 (0.22-0.77)* | 0.45 (0.75-2.76) | - | 0.75 (0.31-1.83) | - | 0.39 (0.15-1.04) | | 0.46 (0.13-1.56) |
| **Binge drinking in the past month** | | | | | | | | | |
| No (Never) | 1 [Reference] | 1 [Reference] | 1 [Reference] | 1 [Reference] | 1 [Reference] | 1 [Reference] | 1 [Reference] | | 1 [Reference] |
| No (Current) | 1.36 (0.99-1.86) | *1.71 (1.19-2.44)* | 0.99 (0.67-1.46) | 1.14 (0.75-1.71) | 1.29 (0.94-1.76) | *1.71 (1.08-2.72)* | *1.51 (1.11-2.04)* | | 1.28 (0.86-1.90) |
| Yes | 0.80 (0.54-1.20) | *0.45 (0.27-0.76)* | 0.57 (0.17-1.90) | - | 0.67 (0.38-1.16) | 0.55 (0.22-1.39) | 0.65 (0.42-1.02) | | 0.64 (0.35-1.15) |
| ****Model 2: Adjusted*** |  | | | |  | | | | |
| **Usual Consumption** | | | | | | | | | |
| Never | 1 [Reference] | 1 [Reference] | 1 [Reference] | 1 [Reference] | 1 [Reference] | 1 [Reference] | 1 [Reference] | | 1 [Reference] |
| Former | 0.77 (0.54-1.11) | 0.76 (0.49-1.17) | 1.50 (0.85-2.67) | 2.38 (1.34-4.25) | 1.00 (0.71-1.41) | 1.13 (0.68-1.87) | 1.05 (0.73-1.50) | | 0.92 (0.57-1.48) |
| <1 drink/day | 1.14 (0.82-1.58) | 1.06 (0.72-1.55) | 1.32 (0.85-2.04) | 1.43 (0.90-2.26) | 1.07 (0.77-1.49) | 1.25 (0.77-2.02) | 1.20 (0.86-1.66) | | 0.90 (0.58-1.39) |
| 1-2 drinks/day | 1.25 (0.84-1.86) | 1.33 (0.85-2.10) | 0.51 (0.18-1.45) | 0.55 (0.18-1.70) | 0.71 (0.42-1.21) | 0.72 (0.33-1.54) | 0.63 (0.34-1.16) | | 0.61 (0.28-1.33) |
| >2 drinks/day | 0.70 (0.43-1.14) | *0.31 (0.16-0.60)* | 0.69 (0.11-4.34) | - | 0.60 (0.24-1.50) | - | *0.32 (0.11-0.87)* | | 0.32 (0.09-1.17) |
| **Binge drinking in the past month** | | | | | | | | | |
| No (Never) | 1 [Reference] | 1 [Reference] | 1 [Reference] | 1 [Reference] | 1 [Reference] | 1 [Reference] | 1 [Reference] | | 1 [Reference] |
| No (Current) | 1.18 (0.85-1.63) | 1.16 (0.79-1.69) | 1.21 (0.79-1.86) | 1.36 (0.87-2.12) | 1.07 (0.77-1.48) | 1.24 (0.77-2.01) | 1.25 (0.90-1.74) | | 0.95 (0.61-1.47) |
| Yes | 0.76 (0.49-1.16) | *0.35 (0.20-0.60)* | 0.77 (0.22-2.68) | - | *0.55 (0.31-0.98)* | *0.36 (0.14-0.95)* | *0.50 (0.30-0.83)* | | *0.43 (0.22-0.85)* |
| Abbreviation: OR indicates odds ratio; -, small sample size; *Adjusted for socio-demographic factors: age, sex, race/ethnicity, education, income and health insurance status; OR <1 is interpreted as decreased odds of having an optimal or average cardiovascular health score; Results in italicized font are statistically significant; p<0.05. | | | | | | | | | |

| **Supplemental Table 4. Multivariable Association Between Alcohol Consumption and Life’s Simple 7 Metrics** | | | | | | | | | | | | | | | |
| --- | --- | --- | --- | --- | --- | --- | --- | --- | --- | --- | --- | --- | --- | --- | --- |
|  | Smoking | | | | Physical Activity | | | | Body Mass Index | | | Diet | | | |
|  | Intermediate vs. Poor | Ideal vs. Poor | | | Intermediate vs. Poor | | Ideal vs. Poor | | Intermediate vs. Poor | Ideal vs. Poor | | Intermediate vs. Poor | | | Ideal vs. Poor |
|  | OR (95% CI) | | | | OR (95% CI) | | | | OR (95% CI) | | | OR (95% CI) | | | |
| **Usual Consumption** | | | | | | | | | | | | | | |  |
| Never | 1 [Reference] | 1 [Reference] | | | 1 [Reference] | | 1 [Reference] | | 1 [Reference] | 1 [Reference] | | 1 [Reference] | | | 1 [Reference] |
| Former | 1.70 (0.64-4.46) | *0.56 (0.42-0.75)* | | | 1.19 (0.94-1.52) | | 1.06 (0.88-1.28) | | *0.80 (0.66-0.97)* | 0.96 (0.77-1.19) | | 0.92 (0.78-1.09) | | | 0.51 (0.23-1.15) |
| <1 drink/day | 1.10 (0.43-2.82) | *0.42 (0.32-0.54)* | | | *1.59 (1.27-2.00)* | | *1.51 (1.27-1.80)* | | 1.07 (0.90-1.28) | 1.22 (1.00-1.50) | | 0.87 (0.75-1.01) | | | 0.73 (0.39-1.39) |
| 1-2 drinks/day | 0.44 (0.12-1.58) | *0.25 (0.17-0.34)* | | | 1.33 (0.93-1.89) | | *1.46 (1.11-1.92)* | | 1.20 (0.92-1.57) | *1.83 (1.37-2.45)* | | 0.88 (0.70-1.09) | | | 1.10 (0.44-2.78) |
| >2 drinks/day | 0.74 (0.21-2.62) | *0.14 (0.09-0.21)* | | | 1.18 (0.73-1.89) | | 0.82 (0.57-1.18) | | 1.11 (0.77-1.61) | 1.19 (0.77-1.83) | | 0.76 (0.55-1.04) | | | - |
|  | Total Cholesterol | | | | | Blood Pressure | | | | | Blood Glucose | | | | |
|  | Intermediate vs. Poor | | Ideal vs. Poor | | | Intermediate vs. Poor | | Ideal vs. Poor | | | Intermediate vs. Poor | | Ideal vs. Poor | | |
| **Usual Consumption** | | | | | | | | | | | | | | | |
| Never | 1 [Reference] | | | 1 [Reference] | | 1 [Reference] | | | 1 [Reference] | | 1 [Reference] | | | 1 [Reference] | |
| Former | 1.19 (0.93-1.54) | | | 1.21 (0.94-1.55) | | *1.30 (1.07-1.58)* | | | *1.26 (1.03-1.54)* | | 1.16 (0.87-1.56) | | | 1.05 (0.83-1.33) | |
| <1 drink/day | 0.87 (0.70-1.09) | | | 0.86 (0.69-1.07) | | 1.18 (0.98-1.42) | | | *1.38 (1.15-1.66)* | | *1.52 (1.15-2.01)* | | | *1.56 (1.24-1.97)* | |
| 1-2 drinks/day | 1.01 (0.72-1.41) | | | 0.84 (0.60-1.18) | | 1.08 (0.83-1.41) | | | 1.23 (0.94-1.61) | | *2.28 (1.44-3.61)* | | | *2.04 (1.37-3.05)* | |
| >2 drinks/day | *0.62 (0.40-0.98)* | | | *0.46 (0.29-0.72)* | | 1.08 (0.75-1.57) | | | 0.72 (0.48-1.08) | | *2.48 (1.30-4.75)* | | | 1.63 (0.91-2.92) | |
| Abbreviation: OR indicates odds ratio; -, small sample size; *Adjusted for socio-demographic factors: age, sex, race/ethnicity, education, income and health insurance status; OR <1 is interpreted as decreased odds of having an optimal or average cardiovascular health score | | | | | | | | | | | | | | | |

| **Supplemental Table 5. Multivariable Linear Regression of the Association Between Alcohol Consumption and Cardiovascular Health** | | | | | | | | |
| --- | --- | --- | --- | --- | --- | --- | --- | --- |
|  | Overall Cohort | Women | | Men | | <65 | | ≥65 |
|  | Β Coefficients | Β Coefficients | | Β Coefficients | | Β Coefficients | | Β Coefficients |
| **Usual Consumption** | | | | | | | | |
| Never | 0 [Reference] | 0 [Reference] | | 0 [Reference] | | 0 [Reference] | | 0 [Reference] |
| Former | -0.02 (-0.18, 0.15) | -0.22 (-0.43, -0.02) | | 0.12 (-0.15, 0.40) | | 0.06 (-0.17, 0.30) | | -0.10 (-0.32, 0.13) |
| <1 drink/day | 0.14 (-0.01, 0.29) | 0.14 (-0.04, 0.32) | | 0.09 (-0.17, 0.35) | | 0.11 (-0.10, 0.32) | | 0.19 (-0.2, 0.40) |
| 1-2 drinks/day | 0.09 (-0.13, 0.30) | 0.39 (0.06, 0.72) | | -0.12 (-0.44, 0.20) | | 0.04 (-0.26, 0.34) | | 0.12 (-0.19, 0.43) |
| >2 drinks/day | -0.84 (-1.16, -0.53) | -0.87 (-1.70, -0.03) | | -0.82 (-1.20, -0.44) | | -0.81 (-1.21, -0.40) | | -0.77 (-1.27, -0.26) |
|  | White | | Chinese American | | Black | | Hispanic | |
| Never | 0 [Reference] | | 0 [Reference] | | 0 [Reference] | | 0 [Reference] | |
| Former | -0.21 (-0.54, 0.11) | | 0.58 (0.18, 0.97) | | -0.09 (-0.39, 0.21) | | -0.002 (-0.33, 0.32) | |
| <1 drink/day | 0.02 (-0.27, 0.31) | | 0.22 (-0.10, 0.54) | | 0.09 (-0.20, 0.38) | | 0.12 (-0.18, 0.42) | |
| 1-2 drinks/day | 0.30 (-0.05, 0.64) | | -0.30 (-1.11, 0.51)) | | -0.62 (-1.08, -0.17) | | -0.32 (-0.85, 0.20) | |
| >2 drinks/day | -0.89 (-1.33, -0.46) | | -1.11 (-2.77, 0.56) | | -0.89 (-1.68, -0.10) | | -0.80 (-1.55, -0.06) | |
| *Adjusted for socio-demographic factors: age, sex, race/ethnicity, education, income and health insurance status; -, indicates small sample size | | | | | | | | |

**References**

1. Lloyd-Jones DM, Hong Y, Labarthe D, et al. American Heart Association Strategic Planning Task F, Statistics C. Defining and setting national goals for cardiovascular health promotion and disease reduction: the American Heart Association's strategic Impact Goal through 2020 and beyond. Circulation 2010; 121: 586-613.

**2.** Ainsworth BE, Irwin ML, Addy CL, Whitt MC, Stolarczyk LM. Moderate physical activity patterns of minority women: the Cross-Cultural Activity Participation Study. *Journal of women's health & gender based medicine* 1999; **8**: 805-813.

**3.** Unger E, Diez-Roux AV, Lloyd-Jones DM, et al. Association of neighborhood characteristics with cardiovascular health in the multi-ethnic study of atherosclerosis. *Circ Cardiovasc Qual Outcomes* 2014; **7**: 524-531.

**4.** Block G, Woods M, Potosky A, et al. Validation of a self-administered diet history questionnaire using multiple diet records. *J Clin Epidemiol* 1990; **43**:1327-1335.

**5.** Mayer-Davis EJ, Vitolins MZ, Carmichael SL, et al. Validity and reproducibility of a food frequency interview in a Multi-Cultural Epidemiology Study. *Ann Epidemiol* 1999; **9**:314-324.
